# Prevalence of and Variables Associated with Syncope-Related Injuries and Fractures in Germany: A Cross-Sectional Study in General Practices

**DOI:** 10.3390/jcm13061566

**Published:** 2024-03-09

**Authors:** Danilo Christian Gümbel, Marcel Konrad, Sarah Krieg, Andreas Krieg, Karel Kostev

**Affiliations:** 1 University Clinic, Justus-Liebig-Universität, 35390 Gießen, Germany; 2 Department of Health and Social, FOM University of Applied Sciences for Economics and Management, 60549 Frankfurt, Germany; 3 Department of Inclusive Medicine, University Hospital Ostwestfalen-Lippe, Bielefeld University, 33617 Bielefeld, Germany; 4 Department of General and Visceral Surgery, Thoracic Surgery and Proctology, University Hospital Herford, Medical Campus OWL, Ruhr University Bochum, 32049 Herford, Germany; 5 Epidemiology, IQVIA, 60549 Frankfurt, Germany; 6 University Clinic of Philipps-University, 35043 Marburg, Germany

**Keywords:** syncope, injury, fracture, osteoporosis, obesity

## Abstract

**Background:** There is a lack of studies investigating the prevalence of syncope-related injuries in a large representative cohort. The aim of this cross-sectional study is to investigate the prevalence of, and variables associated with syncope-related injuries and fractures in a large outpatient population in Germany. **Methods:** The present study used data from the Disease Analyzer database (IQVIA) and included adults with first-time diagnoses of syncope in 1 of 1284 general practices in Germany between 2005 and 2022 (index date). The prevalence of injuries and separate fractures documented on the index date was examined, and the association of demographic and clinical variables with the risk of syncope-related injuries and fractures was studied using multivariable logistic regression. **Results:** A total of 143,226 patients (mean age: 57.1 years, 56.9% female) were included in this study. The proportion of injuries was 10.4% and increased from 6.4% in the age group 18–30 to 15.0% in the age group >80 years. Female sex was associated with a slightly higher risk of injury (OR: 1.09; 95% CI: 1.05–1.13) and fractures (OR: 1.17; 95% CI: 1.07–1.28). Osteoporosis was associated with a higher risk of injury (OR: 1.25; 95% CI: 1.16–1.34) and fracture (OR: 1.53; 95% CI: 1.33–1.76), while obesity was only associated with a slightly increased risk of injury. **Conclusions:** Syncope-related injuries are common among syncope patients. Factors associated with a higher risk of syncope-related injuries, such as female sex, older age, and osteoporosis, can be incorporated into an effective risk stratification and help to improve the outcome of syncope patients.

## 1. Introduction

Syncope is characterized as a brief and temporary loss of consciousness (TLOC) due to transient global cerebral hypoperfusion, with an immediate onset and spontaneous and complete recovery [1,2,3,4]. With a lifetime cumulative incidence of up to 35% [5], syncope is common in the general population and accounts for approximately 1% of all emergency department visits in the U.S. [6], with an average cost of USD 5400 per hospitalization [7].

Although syncope is often considered a benign clinical entity [1,8,9,10], it can be associated with significantly increased mortality [8,11] and significantly reduced quality of life (QoL) for those affected [12,13,14,15]. Further possible and potentially serious consequences of immediate syncopal loss of consciousness include resulting injuries and fractures [3,16], making the condition a significant public health issue. Investigating the prevalence of syncope-related traumatic injuries in a cohort of 346 consecutive patients with recurrent VVS spells, Ammirati et al. found that 27.2% of patients studied had incurred at least one traumatic injury related to syncope, while 8.9% of the cohort even required hospitalization and surgical treatment due to the severity of their syncope-related traumatic injuries [17]. A systematic review of 23 studies by Jorge et al. came to similar conclusions, with a weighted mean injury rate of 33.5% among all VVS patients included in the studies reviewed [3].

However, there is a lack of studies investigating the prevalence of syncope-related injuries in a large representative cohort. Among other things, this is due to the fact that the majority of the studies directly or indirectly addressing the association between syncope and subsequent injury are based in a clinical setting. Consequently, the patient cohorts used for these studies cannot be assumed to be representative of the overall group of syncope patients, as it can be supposed that only a fraction of syncope patients in the overall population make use of higher-threshold services offered by specialized centers and emergency departments [3].

Therefore, the primary aim of this cross-sectional study is to investigate the prevalence of syncope-related injuries and fractures. The secondary aim was to evaluate variables associated with syncope-related injuries and fractures in a large outpatient population in Germany.

## 2. Methods

### 2.1. Database

The present study used data from the Disease Analyzer database (IQVIA), which has already been described in the scientific literature [18]. Briefly, the database contains demographic, diagnosis, and prescription data collected in office-based practices in Germany. The selection of practices included in the Disease Analyzer database relies on multiple variables (i.e., physician age, specialty group, community size category, and German federal state). The database includes around 3000 general and specialized practices in Germany. 

### 2.2. Study Population and Variables

**Figure 1:**
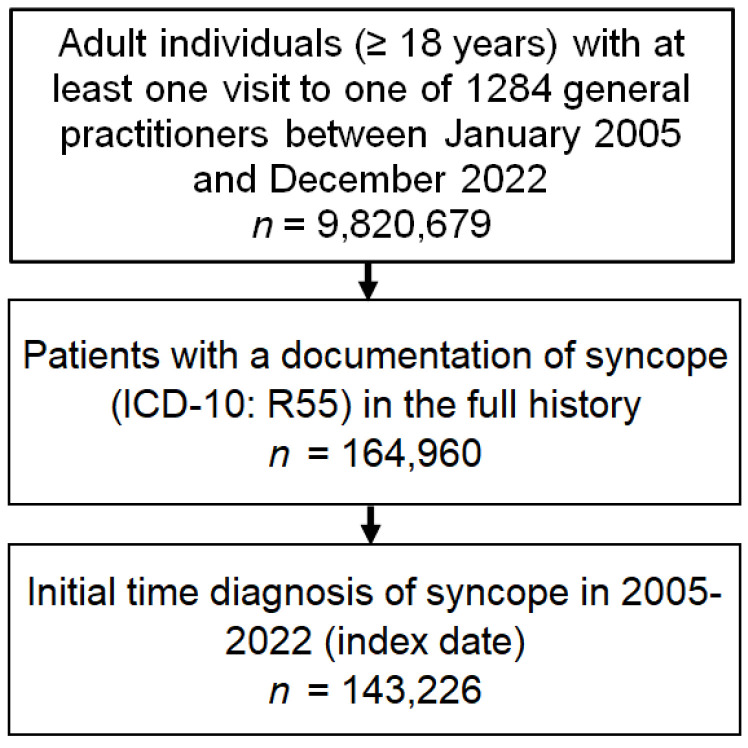
Selection of study patients.

This retrospective cross-sectional study included adults with first-time syncope diagnosis (ICD-10 code: R55) in 1 of 1284 general practices in Germany between 2005 and 2022 (index date) (Figure 1). 

Demographic variables included age and sex. Several chronic conditions frequently associated with frailty and possible increased fall risk were documented within 12 months prior to the index date. These disorders included diabetes (ICD-10: E10–E14], essential hypertension (ICD-10: I10), lipid metabolism disorders (ICD-10: E78), obesity (ICD-10: E66), cardiac arrhythmias (ICD-10: I76–I79), depression (ICD-10: F32, F33), osteoporosis (ICD-10: M81), and dementia (ICD-10: F01, F03, G30).

### 2.3. Statistical Analyses

Baseline characteristics were described using absolute numbers (percentages) for all variables except for continuous age, which was described using the mean (standard deviation). In addition, the prevalence of injuries (ICD-10: S00–T14) and separate fractures (ICD-10: S02, S12, S22, S32, S42, S52, S62, S72, S82, S92, T02, T08, T10, T12) documented on the index date was studied in the overall sample and also within seven age groups (≤30, 31–40, 41–50, 51–60, 61–70, 71–80, >80 years), women and men, and patients with different comorbidities (diabetes, obesity, lipid metabolism disorders, hypertension, cardiac arrhythmias, depression, osteoporosis, dementia).

Finally, the association between these variables and the risk of syncope-related injuries and fractures was studied using multivariable logistic regression adjusted for age, sex, and the comorbidities diabetes, obesity, lipid metabolism disorders, hypertension, cardiac arrhythmias, depression, osteoporosis, and dementia. The results of the models are displayed as odds ratios (ORs) and 95% confidence intervals (95%CI. *p*-values lower than 0.05 were considered statistically significant. All analyses were conducted using SAS version 9.4 (SAS Institute, Cary, NC, USA).

## 3. Results

### 3.1. Baseline Characteristics of the Study Participants

A total of 143,226 patients were included in this study. The baseline characteristics of the study participants are displayed in Table 1. The mean (standard deviation) age was 57.1 (21.6) years, while the prevalence of women was 56.9%. The three most common co-diagnoses were hypertension (33.3%), lipid metabolism disorders (20.0%), and depression (15.2%).

### 3.2. Prevalence of Syncope-Related Injuries and Fractures

**Figure 2:**
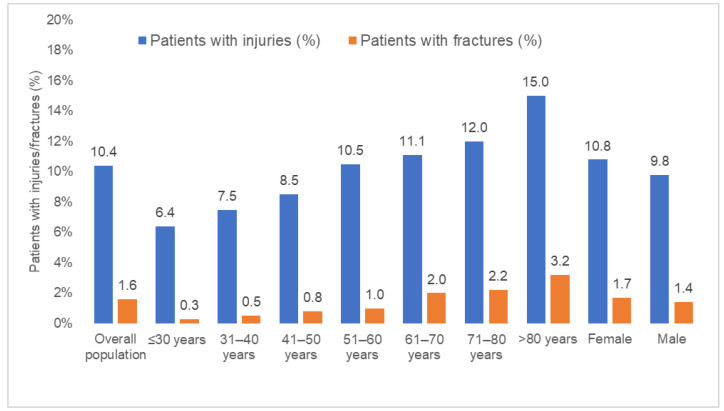
Prevalence of syncope-related injuries and fractures in the overall population and by age and sex.

**Figure 3:**
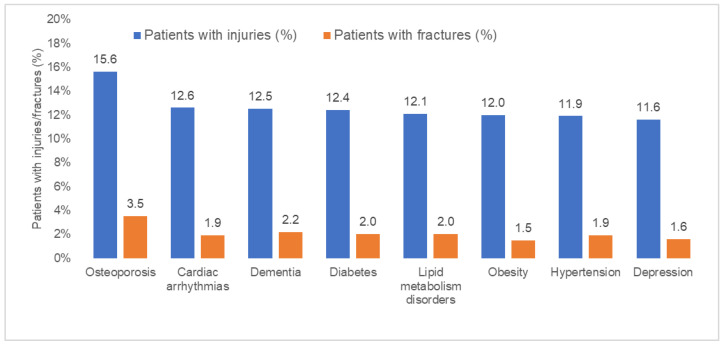
Prevalence of syncope-related injuries and fractures by co-diagnoses.

Figure 2 and Figure 3 displays the prevalence of syncope-related injuries and fractures in the overall population and by age, sex, and co-diagnosis. In the overall population, the proportion of injuries was 10.4% and increased from 6.4% in the age group 18–30 to 15.0% in age group >80 years (Figure 2). This prevalence was highest in osteoporosis patients (15.6%). The prevalence of fractures was 1.6% in the overall population and increased from 0.3% in the age group 18–30 to 3.2% in the age group >80 years. Here too, fractures occurred in 3.5% of osteoporosis patients (Figure 3).

### 3.3. Baseline Characteristics of Patients with and without Injuries and Fractures

Patients with injuries were older than patients without injuries (63.0 vs. 56.5 years, *p* < 0.001), and the proportion of women was slightly higher in the injury group (59.2% vs. 56.6%). Patients with fractures were much older than patients without fractures (70.9 vs. 56.9 years, *p* < 0.001), and the proportion of women was also higher in the former group (62.4% vs. 56.8%, *p* < 0.001). Due to the higher age, the proportions of comorbidities were also significantly higher among patients with injuries and fractures than among patients without fractures or injuries (Table 2).

### 3.4. Variables Associated with Risk of Injury and Fracture

Table 3 shows the results of the univariable logistic regression since Table 4 shows the results of the multivariable logistic regression following stepwise selection. In the univariable regression analysis, all the included variables were significantly associated with an increased risk of injuries. However, in multivariable regression analysis, only higher age (OR: 1.02; 95% CI: 1.01–1.02 per year), female sex (OR: 1.09; 95% CI: 1.05–1.13), osteoporosis (OR: 1.25; 95% CI: 1.16–1.34), and obesity (OR: 1.12; 95%: 1.05–1.20) were associated with an increased risk of injury. Age (OR: 1.04; 95% CI: 1.04–1.04), female sex (OR: 1.17; 95% CI: 1.07–1.28), and osteoporosis (OR: 1.53, 95% CI: 1.33–1.76) were positively associated with a risk of fracture (Table 4).

## 4. Discussion

This cross-sectional study showed that injuries in general were common among outpatients with at least one documented episode of syncope, whereas fractures were rather rare. Furthermore, there was a clear association between the presence of injuries and fractures and older age in the cohort studied. Osteoporosis and female sex were also found to be associated with a more frequent occurrence of injuries and fractures among patients with at least one documented episode of syncope.

Although syncope can be attributed to benign causes in a large number of cases [1,8,9,10], the immediate loss of consciousness is associated with a considerable risk of resulting injuries and fractures [3,17,19]. Both Jorge et al. in a systematic review and Ammirati et al. found markedly higher prevalences of syncope-related injuries among the syncope patients studied, at around one third and one quarter, respectively [3,17]. Investigating the risk of patients hospitalized for unexplained syncope and orthostatic hypotension, Johansson et al. found a fracture prevalence of 27% among the 30,399 individuals observed [20]. Like the results of Jorge et al. and Ammirati et al. discussed above, this represents a clear deviation from the findings of our study. The reason for these differences can be the fact that the studies of Jorge et al. and Ammirati et al. were conducted in hospital settings, where clinically more severe cases of syncope are treated [3], since our study was based on outpatients with probably less severe symptoms.

We found a significant association between the incidence of injuries and fractures among syncope patients and their age. One possible explanation for this association could be the increase in factors associated with older age, which in turn increase the risk of adverse outcomes in syncope, such as the occurrence of an injury or fracture as a result of syncope-related falls. Thus, older age is associated with an increased risk of frailty, as an overarching concept of an often progressive inability to adequately restore physical homeostasis following a stressor [21]. Various changes in the structural composition and metabolism of the bones often result in weaker and more brittle bones with age, which can also cause the general risk of fracture to increase with age [22].

Furthermore, our study showed that women had an increased risk of injury compared to men, particularly for the occurrence of fractures, which is consistent with the findings in the existing literature on this topic. Post-menopausal women in particular have a significantly increased risk of developing osteoporosis, as hormonal development during menopause, especially a lack of estrogen, has an unfavorable effect on bone stability and quality and results in an imbalance between bone formation and bone resorption in favor of resorption [23]. For example, women aged >65 and >80 had a higher risk of suffering a fracture during their lifetime than male patients in the same age groups [24].

A further finding of our study is the positive association between obesity and injuries, but there is no association between obesity and fractures. Individuals with obesity experience restricted mobility, raising the likelihood of falls. For example, Finkelstein et al. reported a strong association between body mass index and the probability of sustaining an injury [25]. Vice versa, not obesity but underweight is a known risk factor for fractures, since obesity is usually not associated with an increased risk of fractures [26,27].

The positive association between osteoporosis and fractures found in our study is well known. Osteoporosis leads to a decrease in bone mineral density, making bones porous and more susceptible to fractures. The loss of minerals, such as calcium, compromises the structural integrity of bones. The weakened bones are more prone to breaking, even with minimal force or trauma [28].

Although this study is based on a large patient population, the most important limitation is the bias created by the study design. By including only outpatients with a first visit after the first diagnosis of syncope, we may have missed many adverse events. For example, a patient only diagnosed with a fracture after seeing their GP would not have been counted. However, the database used does not include hospital data. Secondly, the use of ICD-10 codes might result in the misclassification and undercoding of specific diagnoses. Moreover, ICD-10 codes do not allow any statements to be made about the severity of the injuries and fractures recorded. Thirdly, the present study design does not allow us to differentiate between patients with single syncope episodes and those with recurrent syncope attacks. This would also have been interesting in view of the existing literature, which discusses an increased risk of syncope-related injury and risk of more severe injuries with an increasing number of syncope episodes [17,21]. Fourthly, the database does not include data on mortality. Finally, based on the data available, it is not possible to draw any conclusions regarding causality, and we were therefore only able to make assumptions regarding associations between different factors in this study.

## 5. Conclusions

Syncope-related injuries are common among syncope patients. Factors associated with a higher risk of syncope-related injuries, such as female sex, older age, and osteoporosis, can be incorporated into an effective risk stratification and help to improve the outcomes of syncope patients.

## Figures and Tables

**Table 1:** Baseline characteristics of the study sample.

Variable	Patients with Syncope (*N* = 143,226)
Age (in years)	
Mean (standard deviation)	57.1 (21.6)
≤30	24,399 (17.0)
31–40	13,119 (9.2)
41–50	15,333 (10.7)
51–60	20,603 (14.4)
61–70	19,731 (13.8)
71–80	27,159 (19.0)
>80	22,882 (16.0)
Sex	
Female	81,447 (56.9)
Male	61,779 (43.1)
Co-diagnoses	
Diabetes	19,472 (13.6)
Obesity	9157 (6.4)
Lipid metabolism disorders	28,618 (20.0)
Hypertension	47,622 (33.3)
Cardiac arrhythmias	16,250 (11.4)
Osteoporosis	7036 (4.9)
Depression	21,782 (15.2)
Dementia	6386 (4.5)

Abbreviation: ICD-10 International Classification of Diseases, 10th revision (ICD-10). Data are listed as absolute number (percentage) unless otherwise specified.

**Table 2:** Baseline characteristics of the study patients with and without injuries and fractures.

Variable	Patients with Injuries (%)	Patients without Injuries (%)	Patients with Fractures (%)	Patients without Fractures (%)
*N*	14,860	128,366	2234	140,992
Age (in years)				
Mean (standard deviation)	63.0 (20.3)	56.5 (21.6)	70.9 (15.9)	56.9 (21.6)
≤30	10.4	17.8	2.8	17.3
31–40	6.6	9.5	3.0	9.3
41–50	8.7	10.9	5.6	10.8
51–60	14.6	14.4	11.1	14.4
61–70	14.7	13.7	18.0	13.7
71–80	21.9	18.6	27.3	18.8
>80	29.0	15.2	32.3	15.7
Sex				
Female	59.2	56.6	62.4	56.8
Male	40.8	43.4	36.7	43.2
Co-diagnoses				
Diabetes	16.3	13.3	17.5	13.5
Obesity	7.3	6.3	6.3	6.4
Lipid metabolism disorders	23.3	19.6	25.6	19.9
Hypertension	38.1	32.7	41.2	33.1
Cardiac arrhythmias	13.8	11.1	14.0	11.3
Osteoporosis	7.4	4.6	11.0	4.8
Depression	17.0	15.0	16.0	15.2
Dementia	5.4	4.4	6.4	4.4

**Table 3:** Variables associated with risk of injury and fracture in syncope patients (univariable logistic regression).

Variable	All Injuries		Fractures	
	OR (95% CI)	*p*-Value	OR (95% CI)	*p*-Value
Age (effect per year)	1.02 (1.01–1.02)	<0.001	1.04 (1.04–1.04)	<0.001
Female	1.11 (1.07–1.15)	<0.001	1.26 (1.16–1.37)	<0.001
Male	Reference		Reference	
Diabetes	1.27 (1.21–1.33)	<0.001	1.35 (1.21–1.51)	<0.001
Obesity	1.18 (1.10–1.26)	<0.001	0.98 (0.82–1.16)	0.807
Lipid metabolism disorders	1.25 (1.20–1.30)	<0.001	1.39 (1.26–1.53)	<0.001
Hypertension	1.27 (1.23–1.32)	<0.001	1.42 (1.30–1.54)	<0.001
Cardiac arrhythmias	1.29 (1.22–1.35)	<0.001	1.27 (1.13–1.44)	<0.001
Osteoporosis	1.65 (1.54–1.76)	<0.001	2.34 (2.13–2.79)	<0.001
Depression	1.16 (1.11–1.22)	<0.001	1.07 (0.95–1.19)	0.279
Dementia	1.25 (1.16–1.35)	<0.001	1.48 (1.24–1.75)	<0.001

**Table 4:** Variables associated with risk of injury and fracture in syncope patients (multivariable logistic regression).

	OR (95% CI) *	*p*-Value
All injuries		
Age (effect per year)	1.02 (1.01–1.02)	<0.001
Female	1.09 (1.05–1.13)	<0.001
Male	Reference	
Obesity	1.12 (1.05–1.20)	0.001
Osteoporosis	1.25 (1.16–1.34)	<0.001
Fractures		
Age (effect per year)	1.04 (1.04–1.04)	<0.001
Female	1.17 (1.07–1.28)	<0.001
Male	Reference	
Osteoporosis	1.53 (1.33–1.76)	<0.001

* Multivariable logistic regression adjusted for age, sex, and co-diagnoses. Only variables with significant values are displayed.

## Data Availability

The original contributions presented in the study are included in the article, further inquiries can be directed to the corresponding author.
